# Perception and Longevity Control in Invertebrate Model Organisms—A Mini-Review of Recent Advances

**DOI:** 10.3390/biom15020187

**Published:** 2025-01-28

**Authors:** Nicholas Pontillo, Yang Lyu

**Affiliations:** Department of Molecular Biology and Biochemistry, Rutgers, The State University of New Jersey, Piscataway, NJ 08854-8000, USA; np743@cabm.rutgers.edu

**Keywords:** perception, longevity, lifespan, life history theory, *Drosophila melanogaster*, *Caenorhabditis elegans*

## Abstract

Perception alone can, in some cases, be sufficient to modulate aging and longevity. These influences on aging are perhaps mediated by changes in motivational states that regulate metabolism and physiology to impact health. Simple invertebrate models uniquely enable detailed dissection of integrative pathways linking perceptions to aging and remain the leading systems for advancing this field. Over the past 25 years, studies using the fruit fly *Drosophila melanogaster* and the nematode *Caenorhabditis elegans* have demonstrated that sensory cues, such as those related to food or mating, can influence aging independently of the physical acts associated with them. In this review, we highlight recent advancements in these invertebrate models, focusing on two key areas of progress: (i) the discovery of lifespan modulation driven by novel sensory cues across multiple modalities, including non-sexual social experience, light, and dietary choices; and (ii) the assignment of new aging-regulation functions to specific neurons downstream of sensory perception. The latter offers an exciting first glimpse at the neuronal circuits integrating sensory cues, motivational states, physiology, and aging.

## 1. Introduction

According to life history theory, the rate of aging can be modified by the division of scarce resources between short-term and long-term reproduction—the latter including somatic maintenance and behaviors that tend to increase long-term survival. Part of this division, and therefore, the rate of aging, is set genetically, but another part can be modulated in real time by the organism in response to environmental conditions [1,2,3,4]. Perhaps the most well-studied manifestation of this phenomenon is the promotion of reproductive investments by dietary resources—particularly total calories and protein—and the converse promotion of longevity by food or protein scarcity—although there has been debate over whether and to what extent longevity gains “must” be traded off with worse reproductive or other types of performance [5,6].

As the organism itself can change its physiology to prioritize either short-term reproduction or longevity, chronic sensory perceptions and associated motivational states can powerfully influence the rate of aging. Somewhat counterintuitively, this subject is most well-documented in comparatively simple invertebrate models—the nematode *Caenorhabditis elegans* and the fruit fly *Drosophila melanogaster*. Sensory systems that detect specific environmental cues have been shown to regulate physiology and longevity through neuro-signaling pathways in these species [1,2,3,4]. While this biology of aging is well-documented, the precise mechanisms underlying these processes remain poorly understood.

In this mini-review, we examine recent findings on the perceptive regulation of longevity, focusing on (i) critically evaluating the evidence that perception truly mediates at least part of a reported longevity phenotype, especially for newly reported sensory modalities, (ii) highlighting newly discovered mechanisms and pathways, and (iii) discussing the implications of these findings for classical and evolutionary theories of aging. Aging is a deeply studied research topic, and we limit the scope of our discussion only to longevity phenotypes that may be attributed to “external” perceptual cues such as via olfaction, gustation, vision, and somatosensation.

## 2. Dietary Cues

### Food-Related Cues and Dietary Restriction

Diet is one of the most well-studied variables that influences aging. In particular, dietary restriction (DR)—defined as the restriction of nutrient ingestion to the minimum necessary to prevent malnutrition—is known to extend lifespan in multiple animal models [7]. Studies in *D. melanogaster* and *C. elegans* produced some of the earliest evidence that diet-related sensations can modulate the rate of aging [8,9,10].

**Figure 1:**
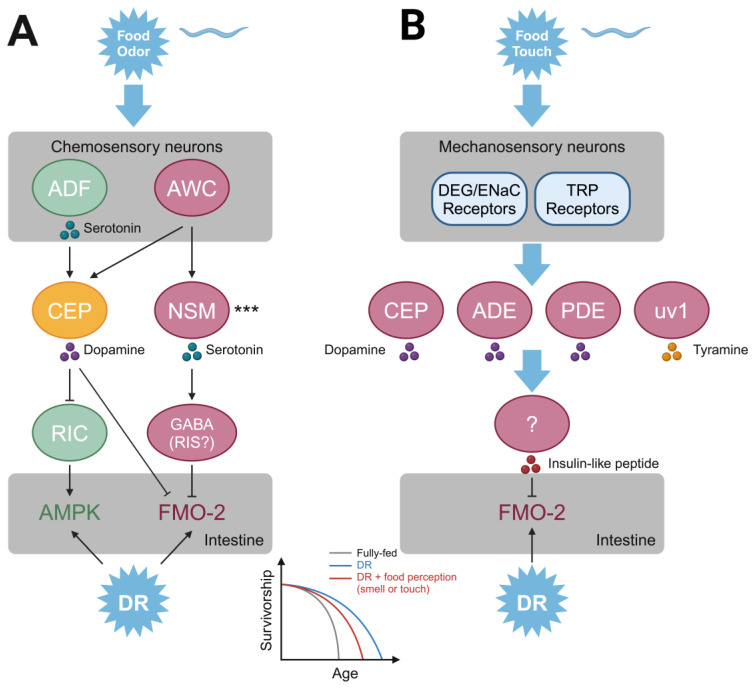
Neural circuits linking food cues and longevity in *C. elegans*. (**A**). Circuit by which food odorant information regulates FMO-2 and/or AMPK activity to abrogate the pro-longevity effect of DR. Pathway components suggested by Zhang et al. [13] are shown in green, while components suggested by Miller et al. [14] are shown in red, and common components are shown in yellow. *** Note that conflicting evidence was presented on whether NSM neurons respond to food odors. (**B**). Summary of the circuit delineated by Kitto et al. [16]. Created in BioRender. Lyu, Y. (2025) https://BioRender.com/k57e037 (accessed on 1 January, 2025).

One such piece of evidence was the finding that exposure to food odors alone is sufficient to abrogate the pro-longevity effect of DR in *D. melanogaster* [11] and *C. elegans* [12]. In *C. elegans*, two recent studies have delineated the neural pathways responsible in remarkable detail [13,14], though with some unresolved differences. Food odorants partially abrogate the effect of DR via a neural circuit involving serotonergic olfactory neurons and downstream dopaminergic and octopaminergic neurons, which ultimately regulate intestinal expression of either the energy sensor adenosine monophosphate-activated protein kinase (AMPK) [13] or flavin-containing monooxygenase 2 (FMO-2) [14]. Commonalities and discrepancies between the models proposed by the two studies are noted in Figure 1.

Beyond chemosensation, food-related tactile stimuli also regulate lifespan. In *C. elegans*, dopaminergic neurons innervate the epidermis with mechanosensitive cilia, which are sensitive to the light tactile stimuli produced by encountering bacteria, their main food source. Activation of these neurons by tactile stimuli putatively induces dopamine release, which inhibits locomotion (the “slowing response” to touched food), presumably to facilitate feeding [15]. A recent study reported that this type of tactile stimulation partially abrogates the pro-longevity effect of dietary restriction by impairing DR-induced changes in insulin and FMO-2 signaling [16]. Thus, both chemosensory and mechanosensory perception of food (or lack thereof) are critical for DR lifespan phenotypes in *C. elegans*.

In line with the unique roles of various nutrients in modulating lifespan and physiology, the perception of different nutrients may likewise have distinct effects. In *D. melanogaster*, we and colleagues found that merely segregating the primary carbohydrate (sucrose) and protein (yeast) sources into separate portions (termed the “choice” paradigm) can either dramatically [17,18,19] or moderately [20] reduce lifespan, though with sex differences. Furthermore, there is evidence that differential positioning of the nutrients can reverse the direction of the effect [21], though this requires validation in a well-controlled environment. Strilbytska and colleagues suggest that flies choosing their own diet may live shorter because of differences in nutrient intake, specifically an increase in consumed protein, which was argued to reflect a fitness-maximizing investment in reproduction at the expense of optimal longevity [20,22]. In contrast, our group’s data suggest that nutrient intake alone does not fully account for the observed effects and that feeding and longevity phenotypes can be uncoupled, suggesting instead that some aspect of nutrient perception in the choice paradigm may shorten lifespan [19]. Nevertheless, discrepancies in measured food intake across these two research groups (perhaps due to differences in the time allowed for the consumption assay) constitute an unresolved issue for future investigation.

Shortened lifespan on the choice diet requires neuronal expression of the type 2A serotonin receptor (5-HT2A) [19], and there is preliminary evidence that 5-HT2B plays a similar role [23] (unpublished). However, the identity of the serotonergic neurons and their downstream, 5-HT2R - expressing partners within the Drosophila nervous system that regulate aging in response to choice remain unknown. 5-HT2Rs are expressed by “F cells”, neurons that innervate the fan-shaped body, a brain region that receives serotonergic input [24] and is involved in nutrient valence and feeding choice [25,26]. They are also expressed in the olfactory system [27], and the *Drosophila* sensory integration center known as the Ellipsoid Body, which is known to regulate longevity in response to externally perceived cues [28].

Additionally, both 5-HT2Rs are expressed in median neurosecretory cells that produce *Drosophila* insulin-like peptides 2, 3, and 5, known as insulin-producing cells (IPCs) [29]. An important advance in the perception–metabolism relationship was recently provided by Yao and Scott [30], who demonstrated that IPCs receive serotonergic input via 5-HT2A from second-order sweet-sensing neurons. Because sweet-sensing gustatory receptor neurons scale their sensitivity to sucrose according to the magnitude of previous chronic sweet taste [31], the serotonergic input to IPCs could conceivably be altered by the chronic consumption of unmixed sucrose in the choice paradigm.

Despite pan-neuronal *5-HT2A* knockdown being sufficient to abrogate the choice phenotype [19], it is possible that it is not an example of aging regulation by external cues per se, but instead that nutrient separation results in shorter lifespan by affecting gut motility, nutrient absorption, and/or gut-derived endocrine signals, consilient with the mammalian role of serotonin in gut peristalsis and nutrient sensing [32,33]. Transcriptomic studies suggest 5-HT2B is, in fact, expressed in *D. melanogaster* enteroendocrine cells (EECs) [34]. *D. melanogaster* EECs also express canonical neuronal markers and gustatory receptors, and are known to regulate longevity in response to the gut’s nutrient contents via neuropeptide F (the *Drosophila* homolog of mammalian neuropeptide Y) signaling to insulin-secreting neurons, ultimately regulating circulating levels of juvenile hormone titer [35]. However, whether the choice phenotype is attributable to adult serotonergic signaling to EECs or other intestinal cells remains to be directly addressed.

How generalizable or translatable is the regulation of lifespan by dietary choice? In humans, dietary choice is the norm, and an emerging market of meal-replacement products (MRPs) is the closest analog to laboratory “fixed” diets. MRPs are known to be effective temporary regimes for achieving weight loss and ameliorating metabolic syndrome [36,37], but long-term use by healthy individuals is not well-studied. Additional research on longevity benefits and the mechanisms behind them in invertebrates, future research in mammalian models, and additional research on long-term usage in humans, would greatly inform the scientific understanding of their true potential as a longevity intervention, and may conceivably justify more widespread adoption of MRPs for general health across the lifespan rather than solely as a weight loss intervention.

## 3. Social Cues

### 3.1. Mating-Related Cues

Various aspects of the social environment are known to influence longevity in *D. melanogaster* and other “non-social” invertebrates [5,38,39]. We focus here on the “external” cue of sex pheromones rather than mating itself and downstream “internal” pathways such as the *Drosophila* Sex Peptide pathway, which have been extensively discussed elsewhere [40].

In *C. elegans*, exposure to male-secreted ascaroside pheromones shortens lifespan of hermaphrodites [41,42] and is especially toxic to other males, although the latter appears to be true only in androdecious species (males and hermaphrodites instead of males and females), possibly as a mechanism to regulate the population sex ratio. Masculinization of the hermaphrodite nervous system is sufficient to sensitize them [43]. The pheromone nacq#1 is synthesized and secreted disproportionately by males, starting at sexual maturity. Nacq#1 is perceived by chemosensory neurons in hermaphrodites, and promotes increased fecundity and reduced lifespan—thus constituting a clear example of a signal of mating opportunity modifying the allocation of resources between reproduction and longevity [44].

In male *D. melanogaster*, exposure to females reduces longevity, and to a greater degree, if it is not followed by mating, which was initially interpreted to reflect a cost of courtship [45]. However, the cost of female pheromone exposure—which also includes reduced triglyceride stores and increased sensitivity to starvation and oxidative stress—can be partially rescued by allowing exposed males to mate either with wild-type [46] or pheromone-masculinized females [47]. Stress and lifespan effects are mediated by neuropeptide F signaling downstream of pickpocket 23-expressing chemosensory foreleg neurons, as well as by insulin-independent changes in *Drosophila* Forkhead Box O (dFOXO) activity and can be modulated by chronic excitation/inhibition of neurons that express the fly homolog of gonadotropin-releasing hormone, Corazonin. Importantly, neither wing damage (suggestive of male-male aggression) nor time spent courting were decreased in males that were exposed but allowed to mate, thus failing to explain the partial rescue of lifespan [47]. These results suggest that in male *D. melanogaster*, the perception of unsatisfied mating opportunities via female pheromone exposure causes physiological stress and shortens lifespan, while actual reproductive resource investments (mating behavior and seminal fluid production) may be largely benign if uncoupled from this stressor.

A recent report provided additional evidence that these stresses are imposed by the nervous system by attributing them to the disinhibition of neuropeptide F target neurons [48]. Additionally, the *Drosophila* homolog of Cholecystokinin (CCK)—Drosulfakinin (DSK)—which is released from a broad set of central neurons including but not limited to IPCs—encodes reward and rescues the effect of female pheromones on lifespan [49]. Interestingly, short-term female pheromone exposure increases subsequent male reproductive competitiveness in mixed-sex housing, suggesting that the costs of exposure are trade-offs for improved fitness [50]. In contrast, the physiological consequences of long-term exposure—if not paired with mating—accentuate the effect of sexual selection by imposing additional fitness costs on unsuccessful males [51].

Thus, female pheromones rather than courtship costs appear to mediate the health effects of female exposure. Pheromones initiate a neuronal response that begins in peripheral chemosensory neurons and likely involves NPF, Corazonin, and DSK signaling, but how pheromone information streams are ultimately integrated into the central brain and affected by these neuropeptides at the circuit level remains unclear.

To what extent are the results in invertebrates extendable to mammals? In mice, neither male nor female odorants affect male lifespan [52], although the presence of female odorants does shorten lifespan when paired with access to additional, different females [53], perhaps reflecting a dosage effect. While mating and/or courtship shorten female lifespan compared to housing with another female [54], exposure to male pheromones does not shorten female lifespan more than living alone [52]. Thus, in “normal” living conditions, opposite-sex pheromones do not have strong effects on lifespan in the mouse, though negative effects on male lifespan reminiscent of invertebrate findings may be present under certain conditions.

### 3.2. Other Social Cues

We have discussed opposite-sex pheromone effects on longevity, but does perceiving same-sex animals also affect health and longevity? In *D. melanogaster*, social isolation is known to extend lifespan compared to co-housing with same-sex vial-mates [55,56,57,58]. In males, this was presumed to result from sperm competition costs. Male *D. melanogaster* react to perceived sperm competition by increasing mating duration [59]. However, it was recently shown that while this behavioral change requires auditory and/or olfactory cues from the rival, they are dispensable for the decrease in lifespan [60]. It should be noted that previously discussed pheromone exposure paradigms compared focal flies to others living in different social environments, but none were alone. Thus, it is possible that isolation extends lifespan by a completely different mechanism (such as increased resource availability) than the relative pro and anti-longevity effects of pheromones from different conspecifics.

Aside from sex, two recent studies have analyzed longevity as a function of conspecific age. Longevity and multiple aspects of health are improved or worsened by co-housing with younger or older same-sex conspecifics, respectively. Co-housing with older conspecifics also decreases resistance to a variety of stresses, though not in all genotypes [61], while the opposite is true in the presence of younger conspecifics [62].

A key question is: What is the mechanism by which conspecifics influence longevity, and to what extent does it involve solely perception? Pheromones from young vial-mates seem to be responsible for extending lifespan, as mutating chemosensory genes in aged flies, or preventing cuticular hydrocarbon (pheromone) production in young flies, both abrogate the pro-longevity effect of young vial-mates [62]. Despite the practice of transferring flies to fresh food every 2–3 days, the fact that *Drosophila* cultures contain a shared food substrate highlights the importance of thoroughly disentangling perceptual or social effects from the transmission of biological factors through feeding. Importantly, a prior study observed that health and longevity improvements can be conferred to otherwise short-lived Cu/Zn superoxide dismutase mutants simply by cohousing with healthier wild-type flies, but pre-exposing the food medium to healthy flies before administering to mutants was not sufficient, validating that transmission of biological factors through food is not necessarily sufficient to explain a robust impact of cohousing on health and aging in fruit flies [63]. Future research could fortify this interpretation by allowing visual and olfactory interaction between separately housed flies.

In summary, co-housing flies with old or young conspecifics exerts largely opposite effects on health and longevity, which may be caused by chemosensory cues rather than transmission of biological factors through shared food. These experiments suggest that, even in non-social insects, social environment may influence health and aging. In contrast, the case for social effects on aging in the context of male–male cohousing (vs isolation) is less strong.

In mice, similar findings on the beneficial effects of younger or healthier conspecifics to the immune function and longevity of otherwise “prematurely aging” individuals have been reported [64], as well as in a Huntington’s Disease model [65,66]. Like in flies, the effect of social interaction, per se, is not clearly distinguished from other consequences of cohabitation, and may, for example, be attributable to differences in shared nest quality [65] or transference of microbiota [64].

## 4. Stress Perception

As stressors in the environment may directly impact an animal’s future fitness, they constitute an important class of signals informing the allocation of resources toward different life history traits. In *D. melanogaster*, exposure to dead conspecifics shortens lifespan via serotonin receptor 2A (5-HT2A). Unlike “necromone”-mediated behavioral responses in social insects [67], this longevity phenotype depends on sight, as exposure in the dark or in visually impaired mutants fails to reduce lifespan. The initial study that characterized this effect reported metabolomic changes in death-exposed flies, which the authors tentatively attributed to a depression or anxiety-like state [68], but the mechanism by which death perception shortens lifespan and its evolutionary significance was otherwise unknown.

The terminal investment hypothesis posits that reproductive effort is inversely related to residual reproductive opportunity [69]. One implication of this idea is that investment in reproduction should plastically respond to changes in environmental conditions that may signal changes in long-term survival prospects. Consistent with this hypothesis, female flies exposed to dead conspecifics display a greater reproductive rate during the exposure period, and this effect can be rapidly reversed upon removal of dead flies [70]. Of note, after measuring fecundity, Corbel and Carazo [70] subsequently tracked longevity, and did not observe shorter lifespan in death-exposed female flies. However, they note that their experimental paradigm only exposed flies to dead conspecifics in early adulthood rather than the whole lifespan. Thus, both studies [68,70] are consistent with death perception acutely shifting organismal resource allocation toward immediate reproduction at the expense of somatic maintenance.

Recently, additional insights have also emerged about the neural signals that mediate health changes in the death perception paradigm. The *Drosophila* Ellipsoid Body is a major sensory integration center within the fly brain and is organized in concentric rings of neuropil. The sight of dead conspecifics activates 5-HT2A^+^ neurons that innervate layers R2, R4d, and R4m. Subsequently, the expression of *Drosophila* insulin-like peptide 3 (*dilp 3*) is increased in the IPCs [28]. Thus, the perception of dead conspecifics likely alters organismal insulin signaling, but how Ellipsoid Body neuron activity is connected to IPCs and regulates Dilp production and/or release is not yet clear.

Like *Drosophila*, *C. elegans* display shortened lifespan and an increase in egg-laying rate after exposure to dead conspecifics. Unlike *Drosophila*, *C. elegans* avoid the corpses of conspecifics (*Drosophila* avoid death-exposed living flies but not corpses themselves [68]). Both the behavioral and longevity phenotypes of *C. elegans* require the function of two olfactory neurons, AWB and ASH, and the detection of the “death signatures” adenosine monophosphate and cysteine rather than visual input, while serotonin signaling is dispensable [71]. Thus, while the perception of dead conspecifics consistently drives a terminal investment phenotype of increased reproductive rate concomitant with reduced lifespan, these effects are mediated by different sensory and neurotransmitter systems.

While death exposure seems to reduce lifespan via the promotion of terminal investment, exposure to other stressors can regulate physiology and aging through other mechanisms. In *C. elegans*, olfactory neurons detect volatiles from pathogenic bacteria and initiate the unfolded protein response (UPR) in the intestines, likely to anticipate proteotoxicity challenges associated with infection. UPR activation depends on cue detection by odorant receptor 3 (ODR-3) and not on an immune response. Chronic exposure and activation of this pathway extend lifespan [72]. In contrast, the regular application of an alarming vibratory stimuli to induce a “flight response” reduces longevity when applied over the lifetime, and transient stimulation worsens subsequent resistance to oxidative stress. These effects are mediated by a neural circuit connecting mechanosensory neurons to tyraminergic RIM neurons, which signal to the intestine to promote insulin signaling, likely promoting the fulfillment of the energetic demands of the flight response while simultaneously inhibiting cytoprotective pathways [73]. In summary, stressor-related perception can have opposite effects on longevity, likely reflecting different organismal response strategies (i.e., terminal investment, stress anticipatory responses, and fight-or-flight stress responses).

## 5. Light

Light exposure has well-established negative effects on invertebrate longevity [74,75,76], particularly via short (blue/ultraviolet) wavelength-induced photooxidative stress and DNA damage [77]. In *D. melanogaster*, lifespan-shortening effects of blue light are not dependent on vision [74], and studies of “stress response” mutants such as superoxide dismutase and sirtuins established that the deleterious effects of light on fly lifespan are attributable to oxidative stress and DNA damage [78]. It is also well-established that light, particularly blue light, is the primary external cue for the entrainment of organismal circadian rhythms in physiology and behavior. Circadian rhythms are driven by an endogenous, cell-autonomous molecular “clock” consisting of a transcription-translation feedback loop of core clock proteins. The phase of the clock is shifted by light exposure [79], and there is evidence that chronic light disruption of circadian rhythms is also harmful (the “circadian resonance” hypothesis) [80,81]. Although discussions of phototoxicity and circadian resonance are outside the scope of this review, a pertinent question is whether there exist any health and longevity effects of light perception or the exercise of vision that can be disentangled from radiative damage and circadian dysregulation.

Two recent studies dissected the effects of monochromatic light on fly lifespan, but obtained different results. In one, daily 12 h exposure to green light extended *D. melanogaster* lifespan even compared to constant darkness. However, the effect was stronger in the *w^1118^* mutant, which lacks eye pigment, and was abrogated by lacing the food with the antibiotic doxycycline, therefore pointing to microbiota and not visual perception as mediators of green light-induced longevity [82]. In contrast, Krittika and Yadav [83], observed the longest lifespan in darkness and progressively decreasing lifespan with 12 h exposure to monochromatic light of decreasing wavelengths, consistent with the notion of direct, wavelength-dependent phototoxicity [83]. Thus, neither study provided evidence that monochromatic light exerts any effect on longevity via a perceptual circuit, and indeed, they obtained contradictory evidence on the effect of green light. Both studies are instead consistent with phototoxicity being the primary driver of effects on health and aging.

Consistent with a previous report [84], a recent study observed an extension of *D. melanogaster* lifespans in constant darkness [85]. However, a surprisingly shorter lifespan could be recapitulated by the daily activation of *Rh1*-expressing photoreceptor neurons to mimic the rhythmic perception of blue light in constant darkness. Circadian rhythm mutants still displayed an extended lifespan in constant darkness, and genetic ablation of the eyes abrogated the effect of light on lifespan. The former shows the independence of the phenotype to circadian rhythms, and the latter to direct phototoxic effects of light. While it is not disputed that at higher light intensities, known phototoxic effects likely dominate, these results nevertheless suggest the existence of uncharacterized pathways linking photoreceptor neuron activity to physiology and aging [85]. Interestingly, it has been argued based on dFOXO mutant research that reduced insulin signaling and increased dFOXO activity occur in constant darkness, and that upregulated dFOXO activity is required for the resulting extension of lifespan [78]. As *D. melanogaster* insulin is released from brain neuroendocrine cells, this hints that the nervous system may regulate a reproduction/longevity trade-off in response to light levels. Directly investigating a potential link between visual exercise and dFOXO should be a goal of future research in this field.

There is evidence that non-circadian, neuronal pathways regulate physiology in response to light in mammals as well as flies, but not in a manner necessarily involving image-forming pathways. In mammals, intrinsically photosensitive retinal ganglion cell (ipRGC) innervation of the suprachiasmatic nucleus (SCN) is the canonical pathway by which light exerts circadian modulation on physiology and behavior. While many non-circadian effects of light on mammalian physiology have been reported (reviewed in [86]), they are mediated either by direct effects on peripheral tissues (such as brown adipose tissue and epidermis) via endogenously expressed opsins, or via non-visual detection by ipRGCs. For example, it was reported that acute thermoregulatory and sleep responses to light in the mouse are mediated by a subset of ipRGCs defined by expression of the *Brn3b* transcription factor, which project widely but not to the SCN, essentially bypassing the circadian master clock [87]. To our knowledge, the exercise of vision per se has not been implicated in longevity regulation in mammals.

## 6. Conclusions and Outlook

There is abundant evidence that sensory perception can modify the rate of aging in animal models, but the levels of understanding of the mechanistic details are starkly different in different sensory modalities and model organisms. The most extensive progress has been achieved in the area of sensory perception of food-related cues, which interface with known “internal” nutrient sensing systems in *C. elegans*. Other prominent research areas include mating and stress-related cues, which are also well-described in both *Drosophila* and *C. elegans*. While particular neurons, genes, ligands, and receptors have been variously identified as critical mediators of the perception-longevity relationship in different cases, more detailed circuit-level understanding often remains to be fully elucidated. The simple anatomy of invertebrate models should render promising the continued identification and characterization of such circuits.

Sensory experience is universal, and its effects on aging, first uncovered through studies in *Drosophila melanogaster* and *Caenorhabditis elegans*, have yet to be rigorously tested in higher organisms. Research using simple animal models offers advantages such as rapid experimentation, genetic access to specific cells, and the ease of uncovering mechanistic pathways, providing initial evidence that inspires the translation of findings to mammals, including humans. However, while highly conserved molecular signaling pathways associated with sensory effects on longevity have been identified in these systems, the regulatory networks that modulate these effects remain poorly understood. Moving forward, investigating how sensory-driven effects function independently of or interact with established longevity pathways—particularly within specific environmental contexts—could significantly advance our understanding of how aging is regulated in real-world conditions.

## Data Availability

Not applicable.
